# Progress in the mechanism and targeted drug therapy for COPD

**DOI:** 10.1038/s41392-020-00345-x

**Published:** 2020-10-27

**Authors:** Cuixue Wang, Jiedong Zhou, Jinquan Wang, Shujing Li, Atsushi Fukunaga, Junji Yodoi, Hai Tian

**Affiliations:** 1 Department of Basic Medicine, Medical College, Shaoxing University, Shaoxing, 312000 China; 2 Division of Dermatology, Department of Internal Related, Kobe University Graduate School of Medicine, Kobe, 650-0017 Japan; 3 Laboratory of Infection and Prevention, Department of Biological Response, Institute for Virus Research, Kyoto University, Kyoto, 606-8501 Japan; 4 Jiaozhimei Biotechnology (Shaoxing) Co, Ltd, Shaoxing, 312000 China

**Keywords:** Drug screening, Drug safety, Molecular medicine

## Abstract

Chronic obstructive pulmonary disease (COPD) is emphysema and/or chronic bronchitis characterised by long-term breathing problems and poor airflow. The prevalence of COPD has increased over the last decade and the drugs most commonly used to treat it, such as glucocorticoids and bronchodilators, have significant therapeutic effects; however, they also cause side effects, including infection and immunosuppression. Here we reviewed the pathogenesis and progression of COPD and elaborated on the effects and mechanisms of newly developed molecular targeted COPD therapeutic drugs. Among these new drugs, we focussed on thioredoxin (Trx). Trx effectively prevents the progression of COPD by regulating redox status and protease/anti-protease balance, blocking the NF-κB and MAPK signalling pathways, suppressing the activation and migration of inflammatory cells and the production of cytokines, inhibiting the synthesis and the activation of adhesion factors and growth factors, and controlling the cAMP-PKA and PI3K/Akt signalling pathways. The mechanism by which Trx affects COPD is different from glucocorticoid-based mechanisms which regulate the inflammatory reaction in association with suppressing immune responses. In addition, Trx also improves the insensitivity of COPD to steroids by inhibiting the production and internalisation of macrophage migration inhibitory factor (MIF). Taken together, these findings suggest that Trx may be the ideal drug for treating COPD.

## Introduction

Chronic obstructive pulmonary disease (COPD) is a slow-developing, incurable lung disease characterised by a sustaining airflow limitation that further develops into common diseases such as pulmonary heart disease and respiratory failure. COPD is caused by a complex interaction between genes and the environment. Cigarette smoking is the leading environmental risk factor for COPD. Fewer than 50% heavy smoker develop COPD,^1^ it indicates that genetics may play a role in regulating the risk of COPD in smokers.^2^ Besides genetics, other risk factors are also involved in the development of COPD, such as age and gender,^3,4^ lung growth and development,^5,6^ exposure to particles,^7–11^ socioeconomic status,^12,13^ asthma and airway hyper-reactivity,^14,15^ chronic bronchitis^12,16^ and infections.^15^ Gender may effect whether a person smoke or experiences certain occupational or environmental exposures; socioeconomic status may be related to lung growth and development, and then influence on susceptibility to developing the disease; and long live will allow greater lifetime exposure to risk factors. Asthma may be a risk factor for the development of COPD. Airway hyper-responsiveness is the second risk factor for COPD, but airway hyper-responsiveness, as an independent predictor of COPD can exist without asthma,^17^ suggesting inflammatory profiles of COPD different from asthmatic subjects. The pathogenesis of COPD remains unclear and has been generally suggested to be related to inflammation, oxidative stress, protease/anti-protease imbalance and decreased immunity.^18^ Smoking, biofuel smoke-induced oxidative stress and excessive protease production are major factors in COPD pathogenesis that cause alveolar cell death, destruction of the extracellular matrix in the alveolar region and loss of alveolar structure.^19,20^ The primary manifestations in the respiratory tract include airway wall remodelling and mucus retention, and further development leads to a serious decline in the lung function.

Currently, the main approach is to deal with symptoms of the airflow limitation caused by the above-mentioned symptoms to improve the resulting dyspnoea through medication, oxygen treatment and rehabilitation therapy. However, there is currently no way to prevent the disease progression. Drug treatment includes bronchodilators and glucocorticoids, with the main types of bronchodilators including the β2 receptor agonists and anticholinergic drugs; however, both have many adverse effects. For example, the main side effects of the β2 receptor agonists are rapid heartbeat, muscle tremors and metabolic disorders.^21^ The side-effects of anticholinergic drugs include dry mouth, blurred vision, urinary retention, postural hypotension, cognitive problems and cardiac rhythm disturbance.^22^ Long-term use of glucocorticoids induces and exacerbates infections, cause hyperglycaemia, osteoporosis and even mental disorders.^23–25^ Therefore, a series of new molecular targeted therapeutic drugs to block COPD progression is under development.

This article introduces the pathogenesis of COPD and pharmacology of related anti-COPD drugs. Specifically, there is a focus on the effective role and mechanism of the small molecule secretory protein thioredoxin (Trx) that is widely expressed in lung tissues such as the type II alveolar cells, macrophages and bronchial epithelium.^26^

## COPD pathogenesis

**Fig. 1:**
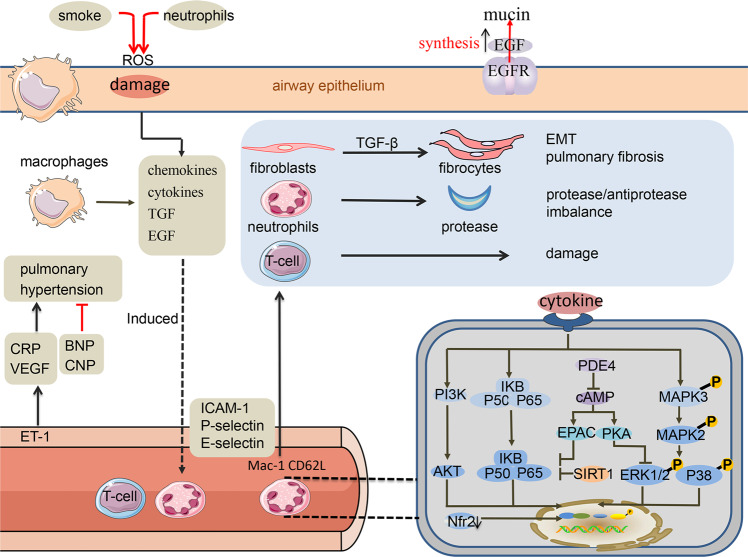
The pathogenesis of COPD is complex and diversified. Oxidative stress may participate in various the pathogenic processes, such as direct injury to lung cells, mucus hypersecretion, inactivation of antiproteases and enhancing lung inflammation through activation of redox-sensitive transcription factors. Under the stimulation of cigarette smoke, pathogen infection and other factors, oxidative stress is induced and the pulmonary inflammatory cells (neutrophils, CD8 T lymphocytes, macrophages) accumulate, resulting in a large number of reactive ROS. The inflammatory cells are activated by the NF-κB, p38MAPK and PI3K signalling. Inflammatory cells (mainly neutrophils) migrate from the circulation to the inflammatory site under sequential regulation involving cytokines and adhesion molecules such as selectin. Proteases are involved in tissue remodelling, inflammation and ECM degradation, thereby participating in the pathological process of COPD. Inflammatory cytokines and chemokines, such as LTB4, IL-8 and TNF-α, and other mediators are secreted into the lungs to aggravate the lung tissue damage and promote inflammatory responses. PDE4 decreases cAMP levels in inflammatory cells and promotes inflammatory cell activity and the release of inflammatory factors. Chronic inflammation stimulates the increase of EGFR and TGF-β1. Activated EGFR is involved in the proliferation of the airway epithelial goblet cells and mucus production. TGF-β1 chemoattracts neutrophils, macrophages and mast cells, and activates PI3K/Akt and/or p38MAPK signalling to induce pulmonary fibrosis and EMT. Endothelin-1 (ET-1) produced by endothelial cells, stimulates the contraction and proliferation of vascular smooth muscle cells and the liver to produce more CRP, and it also induces the synthesis of VEGF. B-type natriuretic peptide (BNP) antagonises renin angiotensin aldosterone system, dilates blood vessels and reduces peripheral vascular resistance, and C-type natriuretic peptide (CNP) dilates blood vessels and inhibits the proliferation of vascular smooth muscle cells

The occurrence and development of COPD is a complex pathological process involving a variety of inflammatory cells, inflammatory mediators and related cell signalling pathways. COPD also regulates the goblet cell proliferation, mucoprotein (MUC) synthesis and mucus secretion. In recent years, molecular biology has revealed new insights regarding the pathogenesis of COPD (Fig. 1).

### COPD and oxidant/antioxidant imbalance

Oxidative stress is an important factor in COPD pathogenesis.^27^ An increased oxidative burden occurs in the lungs of patients with COPD, and oxidative stress may be involved in various the pathogenic processes, such as direct injury to lung cells, mucus hypersecretion, inactivation of antiproteases and enhancing lung inflammation through activation of redox-sensitive transcription factors. COPD patients suffer from oxidative stress caused by the inhalation of cigarette smoke (CS) or harmful substances which causes an accumulation of pulmonary inflammatory cells (neutrophils, macrophages), leading to large numbers of reactive oxygen species (ROS).^28^ Excessive ROS production leads to an oxidative inactivation of anti-proteases, alveolar epithelium damage, increased neutrophil retention and increased expression of various inflammatory mediators in the pulmonary microcirculation, aggravating the development of COPD.^29–31^ In addition, ROS-activated inflammatory cells, now acting as activated inflammatory cells, also generate ROS to further aggravate oxidative stress in tissues. Oxidant-generating systems, such as xanthine/xanthine oxidase, can cause airway epithelial mucus secretion.^32^ Oxidative stress, generated by tobacco smoke and augmented by ROS generated from both inflammatory cells and mitochondrial activity by cells resident in the respiratory tract, regulate mucous cell metaplasia and mucin gene expression such as MUC5AC and MUC5B.^33^ Oxidants are also involved in the signalling pathways for epidermal growth factor, which has an important role in mucus production.^34^ A relative deficiency of anti-proteases, such as α1-antitrypsin, because of their inactivation by oxidants from cigarette smoke or released from inflammatory leucocytes, causes a protease/anti-protease imbalance in the lungs.^35^ Cigarette smoke-induced oxidative stress plays role in enhancing inflammation by regulating redox-sensitive transcription factors, such as nuclear factor kappa-B (NF-κB) and activating protein 1 (AP-1), the extra-cellular signal-regulated kinase (ERK), c-Jun N-terminal kinase (JNK), and p38 mitogen-activated protein kinase (p38MAPK) pathways. ROS also decrease the activity of histone deacetylase (HDAC) which recoil DNA of the histone core to stop transcription,^36,37^ and probably increase the activity of Histone acetyltransferase, which uncoils DNA from the histone core to allow transcription.^38^ This leads to the further recruitment of inflammatory cells, specifically the neutrophils and macrophages in the alveolar spaces. In addition, oxidative stress extends beyond the lung, contribute to several of the systemic manifestations. Peripheral blood neutrophils of COPD release more ROS than in normal subjects and this is enhanced still further in exacerbation.^39^ Products of lipid peroxidation are also increased in plasma in smokers with COPD, particularly during exacerbations, resulting in DNA damage and airway epithelial cell senescence and apoptosis.^39^ In addition, the reactive aldehydes such as acrolein and 4-hydroxy-2-nonenal (4-HNE) formed from lipid peroxidation cause damage to the epithelial cells, acrolein is unstable and toxic, whereas 4-HNE in high concentrations is capable of inducing caspase (a major promoter of cell apoptosis).^40,41^

Nuclear factor E2-related factor 2 (Nrf2) is a transcription factor that regulates many antioxidant genes and plays an important role in the body’s antioxidant stress. Normally, Nrf2 is fixed in the cytoplasm by Kelch-like ECH-associated protein 1 (Keap-1). Under oxidative stress, Nrf2 and Keap-1 dissociate, and Nrf2 is transported to the nucleus, activating the transcription of antioxidant genes.^42,43^ However, the level of Nrf2 in COPD patients is reduced, resulting in a reduction of endogenous antioxidants and weakening the body’s protection against oxidative stress.^44^ Sirtuins 1 (SIRT1) and SIRT6 are also related to the redox state.^45^ SIRT1 is a redox-sensitive protein with a wide range of biological functions, such as inhibition of autophagy, cell aging, emphysema and fibrosis, and inflammation.^43,46,47^ Yao et al. demonstrated that SIRT1 can resist the inflammatory response of cigarette smoke to lung cells caused by oxidative stress.^43^ Inhibition of inflammation may be through deacetylation of NF-κB.^48,49^ SIRT6 is also associated with redox status and inhibits cell aging and fibrosis.^50,51^

### COPD and protease/anti-protease imbalance

The pathogenesis of COPD is closely related to the imbalance of protease/anti-protease, which leads to the destruction of the elastin framework.^52^ Proteases are involved in tissue remodelling, inflammation and degradation of the extracellular matrix (ECM) components and the pathology of COPD.^53,54^ In addition, the products of ECM decomposition, such as collagen and elastin, are themselves chemokines of inflammatory cells and cause persistent respiratory system inflammation in COPD patients, even leading to systemic autoimmune diseases.^55^ Elastase is an enzyme that hydrolyses peptides and other proteins, and there are three main types in lung disease: serine proteases, caspases and matrix metalloproteinases (MMPs). Among them, MMPs are a highly conserved family of endopeptidases, consisting of 28 known members, that depend on zinc and calcium ions. Macrophages and their macrophage-derived MMPs may be the dominant factor in the formation of smoking-related emphysema and COPD. Among the MMPs, MMP-9 and MMP-2 are most involved in emphysema.^56^ MMP-12 plays an influential role in severity of COPD.^57^ MMPs also play a role in vascular remodelling by modulating the migration and proliferation of smooth muscle cells and endothelial cells and mediating the release of the smooth muscle cell mitogens and growth factors, increasing the risk of COPD pulmonary hypertension.^58–61^ The endogenous inhibitors of MMPs are tissue inhibitors of metalloproteinases (TIMPs). TIMPs are a family of low-molecular-weight proteins with four known members.^62^ TIMP-1 inhibits active MMPs, including MMP-1, MMP-3 and MMP-9. It has been reported that changes in MMP-1 poorly correlated with disease intensity and progression in COPD.^63^ TIMP-1 binds to pro-MMP-9 to prevent the activation of pro-MMP-9. However, neutrophil elastase acts by dissociating the binding of TIMP-1 to pro-MMP-9, which allows MMP-3 to activate pro-MMP-9 to become MMP-9.^64^ In serine proteases, neutrophil elastase (NE), cathepsin G and proteinase-3 destroy lung tissue by degrading ECM components, and induces epithelial cells and endothelial cells to release a variety of inflammatory factors to activate and chemoattract neutrophils to produce lung inflammation.^65,66^ Alpha-antitrypsin is a protease inhibitor synthesised by liver cells, which control over the proteolytic activity of NE, proteinase-3, cathepsin G and neutrophils serine protease-4. Evidence from experimental models of emphysema and from individuals with genetic deficiency of alpha-1 antitrypsin provides strong evidence that an imbalance between the enzymes and inhibitors is important in tissue damage and the pathogenesis of COPD.^67^ Protease–anti-proteases imbalance during airway inflammation and airflow restriction may be an important factor affecting airway remodelling and airflow restriction.^68^

### COPD and inflammatory cells, cytokines and chemokines

COPD pathology is characterised by airway remodelling and inflammatory cell infiltration by the neutrophils, CD8 T lymphocytes and activated macrophages.^69^ Neutrophils have been implicated in COPD pathogenesis and the extent of neutrophilic infiltration in lung tissues correlates with COPD severity.^70,71^ Inflammatory factors play a major role in the onset and development of COPD. Under the stimulation of cigarette smoke or other harmful substances, the respiratory tract epithelium secretes inflammatory cytokines and chemokines, such as leukotriene B4 (LTB4), interleukin-6 (IL-6), IL-8 (CXCL8) and tumour necrosis factor-α (TNF-α), and other mediators in the lungs.^72^ These inflammatory mediators aggravate the lung tissue damage and promote inflammatory responses. LTB4 is a lipid mediator derived from arachidonic acid by the sequential action of 5-lipoxygenase (5-LOX), 5-lipoxygenase-activating protein (FLAP) and LTA4 hydrolase (LTA4H) to stimulate leucocyte functions such as cytokines, chemokinesis, lysosomal enzyme release, superoxide anion production, adhesion to endothelial cells, generation of ROS and so on.^73^ IL-6 activates neutrophils, causing neutrophil infiltration at the inflammatory sites, inducing neutrophils to release elastase and various oxygen free radicals, destroying alveolar surfactants, increasing pulmonary vascular permeability and inducing pulmonary oedema.^74^ Activated by IL-8, which is higher in bronchoalveolar lavage fluid (BALF) and sputum of COPD patients.^75^ IL-8 has a similar effect and induces neutrophil migration to the airway and affects degranulation produced by various cell types.^72^ TNF-α stimulates the pulmonary microvascular endothelial cells to promote the accumulation, adhesion and migration of the polymorphonuclear leucocytes by inducing the expression of IL-8 and upregulating endothelial adhesion molecules, causes release of lysosomal enzymes, elastase and large quantities of ROS; and damages the endothelial cells and alveolar epithelium.^76^ TNF-α together with IL-1β has been identified as a key cytokine that is able to initiate inflammatory cascades during exacerbations of COPD. Monocyte chemotactic protein (MCP-1, CCL-2), macrophage inflammatory protein-1α (MIP-1α, CCL-3) are CC-chemokines, which act as chemoattractants for inflammatory cells like macrophages, lymphocytes. Neutrophil-derived CCL-2 and CCL-3 are involved in macrophage recruitment into inflamed tissue.^77^ In patients with COPD, several CC-chemokines like CCL-2, CCL-3 are upregulated to attract specific inflammatory cells, like macrophages, neutrophils and CD8(+) T-lymphocytes into the airway, suggesting the contribution of their respective receptor in the pathogenesis of the disease.^78^

### COPD and adhesion factors

The inflammatory process of COPD is characterised by a continued migration of inflammatory cells (mainly neutrophils) from the blood vessel to the lungs. Neutrophil migration is a carefully regulated series of events involving cytokines and adhesion molecules including the selectins (L-, P- and E-selectin), intercellular adhesion molecule-1 (ICAM-1) and vascular cell adhesion molecule-1 (VCAM-1). The migration has been described as a multistep process including slow rolling, adhesion strengthening, intraluminal crawling and finally paracellular or transcellular migration through the endothelium.^79^ The initial rolling is mediated by L-selectin (CD62L) expressed on neutrophils, and E-selectin and P-selectin expressed on the endothelium. The main ligand for these selectins is the P-selectin glycoprotein ligand (PSGL)-1 (CD162) expressed on neutrophils and certain endothelial cells. Furthermore, E-selectin binds E-selectin ligand 1 (ESL-1) and CD44 on the neutrophil surface, cause the slow rolling of the neutrophil.^80^ Next, firm adhesion is mediated through, for example, the macrophage antigen-1 (Mac-1/CD11b) expressed on neutrophils and its ligand ICAM-1 expressed on the endothelium.^81^ After the neutrophil has been fully arrested, the adhesion of the neutrophil to the endothelial surface is strengthened. Stable binding of ligand to VLA-4 (a4b1-integrin) is rapidly increasing neutrophil infiltration of the inflamed tissue, implicating that internal signals are required for increased adhesion.^79^ The third step, intravascular crawling, involves CD11b and other β2-integrins.^82^ Prior to the final step, transendothelial migration, VCAM-1 and ICAM-1 form the so-called docking structures on the endothelial cells. For the final transendothelial migration, the interaction between two platelet/endothelial cell adhesion molecules (PECAM)-1, expressed at the endothelial cell boundary and on neutrophils, is essential for the ultimate transendothelial migration.^83,84^ Selectin L, E and P have been found in COPD.^85^ PSGL-1 levels were higher in all stable COPD patients than those in in healthy controls.^86^ Increased E-selectin and serum ICAM-1 have also been reported in COPD patients.^87,88^ During migration, the neutrophils release substantial amounts of proteinases and ROS. This process is known as obligate proteolysis and is an important cause for bystander tissue damage in COPD.

### COPD and growth factors

The epidermal growth factor (EGF) is a single-chain polypeptide growth factor that promotes the division of epithelial cells and other cells by binding to a specific epidermal growth factor receptor (EGFR) on target cells to stimulate and maintain a series of cell growth, proliferation and transformation processes. Chronic inflammation increases levels of EGFR and its ligands. The expression and activation of EGFR are positively correlated with the airway epithelial goblet cell proliferation and mucus production. In the airways, activated neutrophils and their secretions play an important role in an EGFR-dependent mucus production. Activated neutrophils secrete TNF-α, which up-regulates the EGFR expression in airway epithelial cells and directly stimulates MUC synthesis.^89^ The expression of EGFR was higher in COPD patients than in smokers with normal lung function, which indicated that COPD was related to the overexpression of EGFR.^90^ EGFR and its ligand EGF binding are the main causes of squamous cell metaplasia, and the growth of epithelial cells is most significant in smokers and COPD.^91^ These indicate that EGFR levels in the small airways of COPD patients were associated with decrease in airway functionality. The transforming growth factor-β (TGF-β) family regulates cell proliferation, differentiation and the extracellular matrix synthesis. TGF-β1 is a chemoattractant for the neutrophils, macrophages and mast cells. TGF-β1 expression is significantly increased in the airway epithelial cells of COPD patients, and an active TGF-β signalling is involved in COPD pathogenesis.^92^ In COPD patients, TGF-β promotes a fibrotic airway remodelling, which can further contribute to a diminished lung function.^93^ Loss of the alveolar parenchymal tissue may be caused in part by an up-regulation of MMP expression in response to TGF-β signalling, leading to an ECM degradation.^94,95^ In COPD, epithelial to mesenchymal transition (EMT) which is associated with airway remodelling and obliteration is activated by canonical pathways such as TGF-β, which induce expression of nuclear transcription factors pSMAD2/3 and reduced inhibitory SMAD6/7 expression.^96,97^ TGF-β also induces and promotes the increased expression of EGF, EGFR and its related signalling pathways, and their synergism induces EMT-related phenotypic changes.^97,98^

### COPD and cAMP

Cyclic adenosine monophosphate (cAMP) is a ubiquitous secondary messenger that regulates a variety of essential processes in diverse cell types via cAMP-dependent effectors such as protein kinase A (PKA) and/or the exchange proteins directly activated by cAMP (EPAC). EPAC and PKA inhibit the human airway smooth muscle induced by a cigarette smoke extract (CSE) by blocking the activation of the NF-κB and ERK, respectively, and by releasing neutrophil chemokine IL-8, which together exert anti-inflammatory effects. cAMP also mediates the airway smooth muscle relaxation.^99,100^ cAMP plays a key role in the functions of many airway cells including controlling ciliary beat frequency (critical for mucus clearance) in airway epithelial cells.^101^ In COPD, increases in cAMP levels, activation of PKA and enhanced protein phosphorylation have the potential to reduce inflammation and immunomodulation, relax airway smooth muscle, inhibit chemotaxis and abnormal release of inflammatory and cytotoxic mediators, and reduce proliferation and migration of inflammatory cells.^102^ Phosphodiesterases (PDEs) are the only way to degrade cyclic nucleotides in the body, thereby ending the biochemical effects conducted by these second messengers (cAMP or cGMP).^103^ In the BALF from COPD patients, cAMP levels are decreased while PDE levels are increased.^104^ These indicate that cAMP is regulated via PDEs, and two direct downstream effectors of cAMP (PKA and Epac).

### COPD and peptide factor

COPD also includes a gradual increase in pulmonary arterial pressure, and 20–91% (depending on the definition, severity and measurement of COPD) have developed pulmonary hypertension.^105^ Endothelin-1 (ET-1) is an effective vasoconstrictor produced by endothelial cells, which can stimulate the contraction and proliferation of vascular smooth muscle cells. The synthesis and secretion of endothelin in patients with COPD increase,^106^ and during the exacerbation of COPD, the level of endothelin further rises and participates in the formation of pulmonary hypertension.^107,108^ ET-1 can also stimulate the liver to produce more C-reactive protein (CRP) by up-regulating IL-6. CRP further stimulates the release of a variety of biologically active substances, such as endothelin-1, IL-6, etc. Amplify the inflammatory effect.^109,110^ In addition, ET-1 can also participate in the pathological process of COPD by inducing the synthesis of vascular permeability factor (VEGF). The family of natriuretic peptides includes A-type natriuretic peptide, B-type natriuretic peptide (BNP) and C-type natriuretic peptide (CNP). BNP and CNP play a major role in the occurrence and development of COPD. BNP antagonises the renin–angiotensin–aldosterone system, dilates blood vessels and reduces peripheral vascular resistance.^111^ CNP also has a strong vasodilator effect and inhibits the proliferation of vascular smooth muscle cells. CNP can block the synthesis of VEGF induced by hypoxia and endothelin at the transcriptional level, thereby inhibiting VEGF-mediated hyperplasia of endothelial cells.^112^ It may play an important role in the development of pulmonary hypertension.

### COPD and the NF-κB pathway

The transcription factor NF-κB is studied in systemic inflammation and has been noticed in COPD patients.^113^ In an inactivated state, NF-κB is located in the cytosol and is complexed with the inhibitory protein inhibitor kappa B (IκB). There are a number of different IκB proteins such as IκBα, IκBβ, IκBγ, IκBɛ and Bcl-3. IκBβ is only phosphorylated by certain stimuli including LPS (lipopolysaccharide) and IL-1β, whereas IκBα phosphorylation is triggered by most NF-κB activators. When a variety of extracellular stimuli act on receptors of the respiratory epithelium, IκB kinase (IKK) is activated. IKK, in turn, phosphorylates IκBα, resulting in ubiquitination and dissociation of IκBα from NF-κB. NF-κB (p50 and p65) is then translocated into the nucleus where it binds to specific sequences of DNA to cause an inflammatory response.^114,115^ NF-κB activates proinflammatory genes encoding cytokines and chemokines, such as IL-1β, IL-8 and TNF-α. The cytokines produced by NF-κB pathway play essential roles in inflammatory cell migration and strengthen oxidative stress during COPD development, further aggravating the condition.^116,117^ Both passive smoking and an intratracheal infusion of LPS induce COPD in rats via the NF-κB signalling pathway.^118^ In respiratory tract biopsies from COPD patients, activated NF-κB levels were significantly higher than that of normal people, while the level of IκB in the lung tissue from smokers or COPD patients was significantly lower than that of non-smoking healthy individuals.^119^ Overexpression of IKK-β in mouse airway epithelial cells results in an increase in inflammatory mediators and neutrophilic inflammation that is reminiscent of the COPD airway following bacterial challenge.^120^ In addition, inhibition of IKK-β in vivo and in vitro reduced TNF-α induced MUC5AC production. This indicates that the NF-κB pathway plays an important role in the occurrence and development of COPD.

### COPD and the p38MAPK

p38MAPKs are members of the MAPK family activated by a variety of environmental stresses and inflammatory cytokines. It seems to be the most effective MAPK, in stabilising, at post-transcriptional level, the mRNAs for cytokines and chemokines relevant to COPD pathogenesis.^121^ p38MAPKs are divided into four subtypes: p38α, p38β, p38δ and p38γ. Different subtypes are expressed in different tissues, and p38α is most abundant in inflammatory cells.^122^ CS, LPS, inflammatory factors and oxidative stress activate the p38MAPK pathway. In the airways and sputum of patients with COPD, p38MAPK was significantly increased, and its activation was related to the severity of COPD.^123^ In addition, the common pathogenic bacteria of COPD, nontypeable *Haemophilus influenzae*, contains cytoplasmic proteins that up-regulate human MUC5AC mucin transcription via a positive p38MAPK pathway and a negative phosphoinositide 3-kinase-Akt pathway.^124^ The activation of p38MAPK associated with the degree of lung function impairment and alveolar wall inflammation.^125^ Bacterial or viral infection is a common trigger for COPD exacerbations, and exposure to LPS induces p38MAPK activation in rat peritoneal macrophages and dendritic cells as well as an increase expression of inflammatory mediators.^126,127^ Glucocorticoid resistance in COPD patients may also be related to the p38MAPK pathway through phosphorylated glucocorticoid receptor (GR) reducing GR translocation into the nucleus and DNA binding, impairing the GR function.^128,129^ The anti-inflammatory effects of corticosteroids are mediated by GR, and functional impairment of GR is an important mechanism of glucocorticoid resistance.^130^

### COPD and the PI3K/Akt pathways

The phosphatidylinositol 3 kinase (PI3K) pathway is a major pathway regulating cell growth, proliferation, metabolism, survival and angiogenesis. Serine/threonine kinase (Akt), also known as protein kinase B, is an enzyme consisting of a PH domain, a kinase catalytic domain, and a regulatory domain that covalently attaches ATP-phosphate groups to the serine/threonine of protein substrates to alter target protein activity.^131^ Activated PI3K phosphorylates phosphatidylinositol diphosphate to produce the secondary messenger phosphatidylinositol 3,4,5-triphosphate, which binds the PH domains of Akt and phosphoinositide dependent kinase-1. Akt undergoes a conformational change and is transferred to the plasma membrane, leading to Akt activation.^132^ Activated Akt regulates cellular functions by phosphorylating various downstream factors including enzymes, kinases and transcription factors.^133,134^ Dysregulation of Akt activity impacts on all these essential cellular processes, such as cell growth, survival and inflammation.^135^ The PI3K/Akt signalling pathway plays an important role in COPD by regulating inflammatory cell activation, inflammatory mediator release and airway remodelling.^136^ The regulation of PI3K/Akt pathway in neutrophil restore some key COPD neutrophil responses.^137^ PI3K/Akt pathway regulates macrophage Polarisation in emphysematous mice generated by CS exposure combined with intraperitoneal injection of CSE.^138^ ECM proteins promoted proliferation, migration and adhesion of airway smooth muscle cells form rat models of COPD through activation of the PI3K/AKT signalling pathway.^139^ ROS activates PI3K, initiating the PI3K/Akt signalling pathway. PI3K/Akt pathway plays an important role in the activation of Nrf2, which regulates oxidative stress and inflammation in COPD.^140^ The persistent airway and lung inflammation of COPD is related to a decreased histone deacetylase activity (mainly HDAC2) caused by oxidative stress. Up-regulation of PI3Kδ/Akt signalling reduces the HDAC activity, promoting the transcriptional activation of inflammatory genes.^141^ Another study found that down-regulation of HDAC2 is associated with glucocorticoid resistance.^142^

## New molecular targeted drugs

**Fig. 2:**
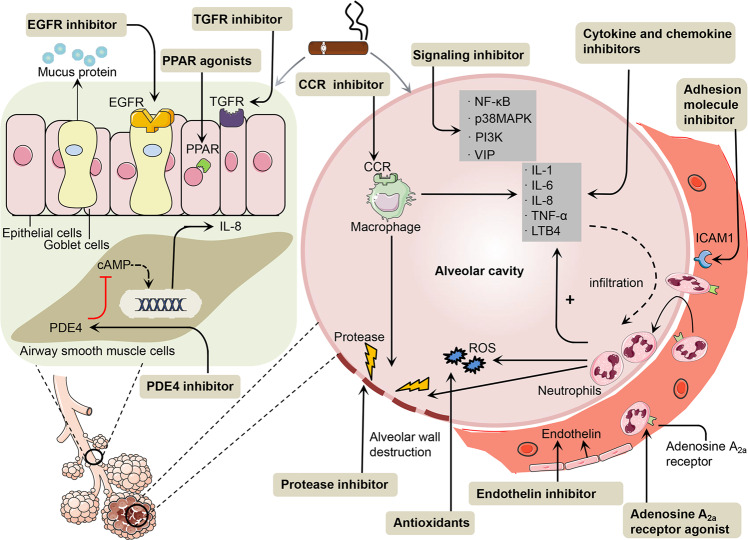
New molecular targeted drugs. Based on the molecular mechanism of COPD, many new molecular targeted drugs have been developing in recent years. Antioxidants scavenge ROS and inhibit oxidative stress in the lungs and reduce cellular damage and inflammation. Protease inhibitors restore the balance between protease and anti-protease by inhibiting. proteases. Cytokine and chemokine inhibitors play an important role in reducing the inflammatory response. Adhesion molecule inhibitors can block inflammatory cells, which continuously migrate from the blood vessels to the tissue. PDE4 inhibitors inhibit PDE4 production to increase the cAMP activity in cells. In the occurrence and development of COPD, the signalling molecules, such as NF-κB, MAPK, PI3K and VIP help regulate inflammation and airway remodellings, and represent plausible targets for the development of therapeutic candidates. Candidate drugs include inhibitors of p38MAPK, NF-κB and PI3K, and vasoactive intestinal peptide (VIP). The inhibitor of EGFR reduces internalisation of EGFR but does not reduce mucin stores. TGF-β inhibitor reduces a fibrotic airway remodelling and downregulates MMP expression, Endothelin inhibitors prevent the progression of pulmonary hypertension in COPD. Adenosine A_2a_ receptor inhibits neutrophil superoxide production, phagocytosis, adhesion and cytokine release. Macrolides reduces the inflammation of COPD by regulating the PI3K/Akt-Nrf2 pathway and control transcription factors such as NF-κB and AP-1 to inhibit the production of inflammatory cytokines. PPAR agonists exert antioxidant and anti-inflammatory effects by down-regulating NF-κB and other pro-inflammatory transcription factors

As the inflammatory signalling pathways closely associated with COPD development have been elucidated, most candidate therapeutic drugs developed in recent years are molecular drugs targeting these signal transmitting substances. The following sections outline new COPD treatment strategies (Fig. 2).

### Antioxidants

Oxidative stress plays a crucial role in the pathogenesis of COPD. In the exhaled breath condensate of COPD patients, the levels of oxidative stress markers such as hydrogen peroxide (H_2_O_2_) and 8-isoprostaglandin (8-IP) are significantly increased.^143^ Through in vivo experiments, antioxidants have been shown to inhibit COPD onset. N-acetylcysteine (NAC) and other glutamines have been clinically tested in this regard. Despite evidence of the beneficial role of NAC in COPD, its therapeutic efficacy in clinical management of COPD has remained controversial due to its reduced bioavailability in an oral form and its acidic nature prohibiting its use in an inhaled form.^144^ In addition, the lack of assurance that there is an effective concentration of glutathione in the lung is another major reason for not achieving the desired therapeutic effect.^145^ Other antioxidant enzymes, such as superoxide dismutase and glutathione peroxidase, have good anti-inflammatory effects on smoking-induced lung inflammation in animal models, and clinical trials are underway.^146^ COPD is also affected by oxidative stress and nitrosative stress. Therefore, nitric oxide synthase inhibitors being developed for acute diseases may also be suitable to treat COPD.

The antioxidant transcription factor Nrf2 downregulates inflammation-associated production of ROS and reactive nitrogen species. Nrf2-deficient mice exposed to CS had more extensive emphysema and more pronounced airway inflammation than wild-type mice.^147^ As a small molecule Nrf2 activator, sulforaphane increased the gene expression of Nrf2, reduced the level of ROS, and prevented the damage of CSE-treated alveolar epithelial cells.^147^ The natural product sulforaphane activated Nrf2 in alveolar macrophage isolated from COPD patients denitrify HDAC2 and restore the sensitivity to the glucocorticoid dexamethasone in a glutathione-dependent manner.^148^ Unfortunately, sulforaphane applied for 4 weeks to patients with COPD did not induce the expression of Nrf2 genes or have an effect on oxidative stress, airway inflammation or lung function.^149^ Resveratrol is a natural SIRT1 activator. In the rat model of COPD treated with resveratrol, serum IL-6 and IL-8 levels were decreased and lung inflammation was inhibited.^150^ In addition, resveratrol activation of SIRT1 also downregulates the activity of MMP9 in fibroblasts in COPD.^151^ In recent years, it has been found that SRT1720 is a compound that can activate SIRT1, which can improve lung function and reduce lung damage caused by CS^152^ (Table 1).

**Table 1:** Development of antioxidants and protease inhibitors for COPD

Drug	Mechanism/effect	Clinical progress	Reference
N-acetylcysteine (NAC) /glutamines	Suppressing oxidative stress	DBPCRT PANTHEON trial (1 year) found 600 mg bid NAC reduced the degree of deterioration in GOLDII-III COPD patients (Chinese Clinical Trials Registry TRC-09000460), Another study (NCT01136239) also found that it was reduced only in high-risk patients, and an improvement in airway function was also observed. However, some low-dose studies (600 mg/day) found no benefit (NCT00184977; the rest are not registered).	^326,327^
SOD/GPx	Reduce ROS	SOD and glutathione peroxidase GPx have good anti-inflammatory effects on smoking-induced lung inflammation in animal models, and clinical trials are underway	^146^
Sulforaphane	Increase the gene expression of Nrf2, downregulates inflammation-associated production of ROS and reactive nitrogen species (RNS)	Sulforaphanetrial (4 weeks) in COPD patients did not induce the expression of Nrf2 genes or have an effect on oxidative stress, airway inflammation, or lung function (NCT01335971).	^149^
Resveratrol	SIRT1 activator	Resveratrol (12 weeks) in COPD patients is under wayb (NCT03819517). A comprehensive assessment of cardiovascular health will be conducted.	^328^
SRT1720	SIRT1 activator	SRT1720 could protect against AECII apoptosis in rats with emphysema and thus could be used in COPD treatment.	^152^
AZD1236	Anti MMP-9 and MMP-12	AZD1236 (6 weeks) in moderate- to-severe COPD patients did not reach statistical significance or have effect on COPD clinical symptoms.	^329^
Sivelestat (ONO-5046)	Protect the lung from NE-mediated tissue damage and control the exuberant inflammatory response	Japan approved ONO-5046 for the treatment of ALI and ARDS. However, many countries have not approved Siveles for clinical use, due to the uncertainty of the randomised double-blind trial results.	^157^
AZD9668	Protect the lung from NE-mediated tissue damage and control the exuberant inflammatory response	AZD9668 (12 weeks) combined with budesonide/formoterol has no effect on lung function, quality of life and lung function in COPD patients	^158^

### Protease inhibitors

Regulating the imbalance between proteases and their inhibitors has attracted much attention as a treatment for COPD. MMPs which degrade elastin are a target for drug development. Non-specific MMP inhibitors are mainly developed as anticancer agents, but they have been reported to cause side effects such as arthritis, so long-term use as a COPD treatment drug may have hidden dangers.^152^ MMP-9 is a member of the MMP family and its endogenous inhibitor is TIMP-1, which binds the carboxyl terminal of the catalytic centre of MMP-9 to form an enzyme-inhibitor complex through noncovalent bonding.^153^ Both are key enzymes regulating ECM degradation and synthesis, and changes in their concentrations are closely related to an airway inflammation damage.^153^ Through culturing BALF cells of COPD patients with healthy individuals, it was found that MMP-9 release by macrophages in COPD patients was significantly increased while healthy control macrophages released more TIMPs.^154^ Selective inhibitors of MMP-9 are also being developed and have proven to be effective in treating COPD in animal models, but have not been effective in clinical trials of COPD.^155^

NE inhibitors are also a valuable potential therapeutic drug, which can not only protect the lung from NE-mediated tissue damage, but also control the exuberant inflammatory response.^156^ Japan approved Sivelestat (ONO-5046) for the treatment of ALI and ARDS. However, many countries have not approved Siveles for clinical use, due to the uncertainty of the randomised double-blind trial results. Other promising NE inhibitors have also been stopped for various reasons.^157^ AZD9668 (12 weeks) combined with budesonide/formoterol has no effect on lung function, quality of life and lung function in COPD patients^158^ (Table 1).

### Chemokine inhibitors and cytokine inhibitors

The levels of IL-1β, IL-6, TNF-α and IL-8 are significantly increased in the sputum and serum of COPD patients.^116^ They may also be a therapeutic target for COPD. As a human anti-IL-1β monoclonal antibody, Canakinumab (45-week treatment) showed no statistical analysis provided for lung function changes.^159^ The receptor inhibitor tocilizumab is an available IL-6 inhibitor with confirmed efficacy in rheumatoid arthritis, but clinical trials in COPD require further study. Endogenous TNF-α plays an important role in the development of pulmonary fibrosis^160^ and causes a secondary interstitial lung disease. As anti-TNF-α therapies, the TNF-α antibody (infliximab) and soluble TNF-α inhibitor (etanercept) have been used as clinical drugs, mainly to treat inflammatory diseases such as rheumatoid arthritis; and the same clinical dose of the TNF-α antibody used in rheumatoid arthritis has a definite effect on bronchial asthma, but has not been confirmed in COPD. In contrast, COPD patients had significantly increased incidence of airway tumours and lung infections caused by TNF-α antibodies,^161^ presenting a difficult problem to be overcome with future anti-TNF-α treatments. Inhibitors of the IL-8 receptor CXC chemokine receptor 2 (CXCR2) effectively block neutrophil infiltration in the lung tissue in animal models and clinical trials.^162^ CXCR2 inhibitors block neutrophil invasion and inhibit mucus production and airway remodelling. However, it should be noted that CXCR2 inhibition triggers side effects similar to those exhibited by the use of glucocorticoids, such as promoting bacterial/fungal infection and delaying wound healing.^162^ Currently, there are clinical trials in progress for the CXCR2 receptor antagonists in COPD, bronchial asthma and cystic fibrosis. CCR1 has an affinity for multiple chemokines, including (MIP-1α/CCL3) and regulated on activation, normal T-cell expressed and secreted (RANTES/CCL5), which are both elevated in lungs of COPD patients.^163,164^ CCR1 antagonists have been developed and are in clinical trials for autoimmune diseases.^165^ CCR2 which is the only receptor for (MCP-1/CCL2 recruits inflammatory cells to lungs in COPD, and increases synthesis of MUC5AC and MUC5B.^166^ The levels of both CCR2 and MCP-1/CCL2 mRNA were increased in bronchial epithelium of COPD patients.^167^ MCP-1/CCL2 production upon cigarette smoke exposure were increased in a mouse model of COPD,^168^ CCR2 inhibitors for COPD have been studied, but statistical analysis not released.^169^

LTB4 is elevated to some extent in the BALF, sputum, serum and lung tissues of COPD patients, and is positively correlated with the COPD severity.^170^ Several inhibitors are under development, one of which has entered clinical trials, but no effective anti-inflammatory effect has been demonstrated.^171^ In recent years, a 5-LOX inhibitor upstream of LTB4 has been under development for bronchial asthma, but current 5-LOX inhibitors have problems such as lack of selectivity, structure–activity relationships, methaemoglobin formation, and poor efficiency and oral availability.^172^ It should be noted that inhibiting LTB4 biosynthesis at the level of 5-LOX or FLAP removes the LTA4 intermediate. However, the latter molecule is an important intermediate in the biosynthesis of anti-inflammatory lipoxins, potentially reducing the net anti-inflammatory effect. In addition, some LTA4H inhibitors are also being developed^173,174^ because these have been suggested to block LTB4 production while preserving LTA4, allowing shunting into lipoxin A4. This features may make LTA4H inhibitors superior therapeutic molecules as compared with 5-LOX or FLAP inhibitors^175^ (Table 2).

**Table 2:** Development of cytokine and chemokine receptor inhibitors for COPD

Drug	Mechanism/effect	Clinical progress	Reference
Canakinumab	Inhibition of IL-1β	A phase I/II RDBPCES of canakinumab (45 weeks), no statistical analysis provided for lung function changes	^159^
Tocilizumab	Inhibition of IL-6	Tocilizumab has efficacy in rheumatoid arthritis, but clinical trials in COPD require further study.	^330^
Infliximab	Inhibition of TNF-α	Infliximab (6 months) did not have clinical benefit but toxicity—higher rate of pneumonia and malignancies (NCT00056264)	^331–333^
etanercept	Inhibition of TNF-α	Etanercept (90 days) is no better than prednisone in the treatment of COPD deterioration(NCT00789997).	^334^
AZD4818	Inhibition of CCR1	AZD4818 (4-week treatment) provided no significant benefit to COPD patients (NCT00629239).	^335^
AZD2423	Inhibition of CCR1	AZD2423 (28-day treatment) in DBPCRT (NCT01215279); study has completed but statistical analysis not released.	^336^
Navarixin (MK-7123)	Inhibition of CXCR2	MK-7123 (6 months) in DBPCRT showed improvement in FEV1 (NCT01006616 and NCT00441701).	^75,336^
BIIL 284	Inhibition of LTB4 receptor	BIIL 284 (12 weeks of treatment) assessed the effects of lung function, exercise tolerance, sputum and safety in patients with COPD (NCT02249247); a 14 day study assessed the impact of biomarkers (NCT02249338)—the results of both studies have not been published. Other LTB4 receptor antagonists have not shown beneficial results	^337^
Zileuton	Inhibition of 5-LO	Zileuton (14 days) in DBPCRT reduced urinary LTE4 levels in hospitalised COPD patients with acute exacerbations but did not significantly in treatment (NCT00493974).	^338^

### Adhesion molecule inhibitors

In the inflammation process of COPD, selectins are essential for the migration of inflammatory cells from the bloodstream into the pulmonary tissue. Therefore, targeting these molecules may inhibit the inflammatory process of COPD. Although several selectin inhibitors have been tested in clinical trials for bronchial asthma patients, they have not proven to be effective.^176^ Bimosiamose, a pan-selectin antagonist, blocks the adhesion of neutrophils, eosinophils and lymphocytes in vitro, and has anti-inflammatory effect in animal models of lung inflammation. Bimosiamose inhalation has good safety and tolerance for COPD patients. It reduces the levels of IL-8 and MMP-9 in the sputum and reduces the number of neutrophils, reduces airway inflammation and improves the lung function,^177^ thus warranting further testing. EL246 is an anti-selectin monoclonal antibody that recognises specific positions on the E and L selectins to inhibit cell adhesion. EL246 is currently being developed as a therapeutic drug for acute exacerbation of COPD.^178^ It is clear that further studies are required to demonstrate the true clinical benefits of bimosiamose and EL246 in COPD patients. Because E, L, P selectins recognise and bind to epitopes containing the carbohydrate sLe^x^ of glycoprotein or glycolipidon on a cell surface. A clinical study of 2-O, 3-O desulfated heparin (ODSH or PGX-100) which is a carbohydrate-based drug was performed in Phase II for COPD. However, the trial has been terminated because the interim analysis showed that ODSH had no effect on the safety of patients with acute exacerbation of COPD^177^ (Table 3).

**Table 3:** Development of other drugs for COPD

Drug	Mechanism/effect	Clinical progress	Reference
Bimosiamose	A synthetic pan-selectin antagonist that targets E-, P- and L-selectin. In vitro, bimosiamose blocks adhesion of neutrophils	Bimosiamose (TBC 1269) was in Phase II for treatment of asthma(inhaled), reperfusion injury (injectible) and psoriasis (topical). There was an additional inhaled version of TBC 1269 in preclinical investigation for asthma. Revotar Ag (Germany) under a license from Encysive is continuing development of an inhaled version of TBC 1269 for asthma and COPD and a cream and subcutaneous administration for psorias (NCT01108913).	^255^
Eleuquin (EL246)	Anti-E/L-selectin monoclonal antibody, which recognises specific positions on the E and L selectins to inhibit cell adhesion.	EL246 (Eleuquin) is under predevelopment by LigoCyte for the treatment of acute inflammatory conditions such as COPD, ischaemic reperfusion injury and transplant reject.	^177^
BIBW 2948	Reduce internalisation of EGFR	Inhalation of BIBW 2948 (4 weeks) reduced internalisation of EGFR but did not reduce mucin stores (NCT00423137).	^179^
PDE4 inhibiotr	Inhibit PDE4 and increase cAMP levels in inflammatory cells, regulating the activity of inflammatory cells, and regulating the release of inflammatory factors to exhibit anti-inflammatory effects	Roflumilast has been approved by the Food and Drug Administration (FDA) as a COPD treatment. Roflumilast relieves the symptoms of dyspnoea in COPD patients and reduces the frequency of acute attacks, but has side effects such as nausea, vomiting, and headache. GSK-256066 (4-week inhaled treatment) in DBPCRT (NCT00549679) improved residual volume and showed no significant trend of FEV1 after bronchodilator. CHF6001 is in clinical testing (28-day treatment) (NCT01730404) but no results have been reported. The others are in clinical testing, such as, MK-0359 (NCT00482235); MK-0873 (NCT00132730); tofimilast (NCT00219622); UK-500,001 (NCT00263874); tetomilast (OPC-6535) (NCT00874497), terminated, (NCT00917150); oglemilast (NCT00671073); QAK423A (NCT01197287); and TPI 1100 (NCT00914433).	^191,194–198^
Bosentan	Blocks endothelin receptor.	Bosentan (18 months) can alleviate the condition of COPD patients and prevent the progression of pulmonary hypertension. This effect is more significant in GOLD III and IV patients. But for COPD patients without pulmonary hypertension, bosentan will aggravate their hypoxaemia.	^199,200^
Solithromycin	Decrease the production of proinflammatory cytokines and chemokines by epithelial and immune cells	A macrolide antibiotic. No data of Solithromycin (28 days) collected for this Outcome due to early termination of the trial (NCT02628769)	^339^
PPAR agonists	Regulates function of multiple cells of the immune system.	PPARγ agonists includes Troglitazone, Rosiglitazone, and Pioglitazone. PPARα agonists includes Clofibrate and Fenofibrate. Patients who took more than two thiazolidinediones (97.1% rosiglitazone) had significantly less COPD deterioration than patients receiving other diabetes drugs. Results information of clinical trail (February, 10 months) has been submitted to ClinicalTrials.gov by the sponsor or investigator, but is not yet publicly available on ClinicalTrials.gov (NCT00103922)	^230,340^

### EGFR and TGF inhibitor

EGFR regulates mucin stores in airway epithelium, which are significantly increased in COPD. BIBW 2948 is the inhibitor of EGFR, and inhalation of BIBW 2948 (4-week treatment) reduced internalisation of EGFR but did not reduce mucin stores.^179^ Inhibition of TGF-β1 signalling may also be a useful therapeutic strategy in COPD. The bone morphogenic protein and activin membrane-bound inhibitor (BAMBI) is a transmembrane glycoprotein, which acts as a negative regulator of TGF β signalling.^180^ BAMBI is induced by members of the TGF family-²-catenin, SMAD3 and SMAD4191, acting as a pseudoreceptor.^181^ BAMBI expression as significantly stronger in COPD patients and that increased plasma BAMBI levels in COPD patients displayed excellent correlations with enhanced plasma TGF-β1 levels.^182^ The Because the mechanisms regulating BAMBI expression are poorly understood, The clinical trail of BAMBI is not developed.^183^ In addition, Small molecule antagonists which inhibit TGF-β receptor kinase or TGF-β activated pathways were studied,^184,185^ although the long-term safety of such drugs might be a problem, particularly as TGF-β affects tissue repair and is a potent anti-inflammatory mediator (Table 3).

### PDE4 inhibitors

PDE4 inhibitors have a selective inhibitory effect on PDE4 specifically expressed in inflammatory, airway smooth muscle and epithelial cells.^186^ Its main biological effects are selective inhibition of PDE4, leading to increased cAMP levels in inflammatory cells, regulating the activity of inflammatory cells and regulating the release of inflammatory factors to exhibit anti-inflammatory effects. They have been developed as new anti-inflammatory drugs for COPD and asthma since the 1980s.^187^ The PDE4 inhibitor roflumilast has been approved by the Food and Drug Administration as a COPD treatment. It was shown roflumilast reduced the diffuse emphysema induced by CS in mice as compared with that in the control group.^188^ Other studies have found that roflumilast inhibits bleomycin-induced pulmonary fibrosis in rats and reduces pulmonary vascular remodelling.^189,190^ Clinical trials have proven that roflumilast relieves the symptoms of dyspnoea in COPD patients and reduces the frequency of acute attacks.^191^ Once-daily administration of roflumilast significantly improves forced expiratory volume in 1 s and decreases exacerbations, particularly in patients with severe disease,^192,193^ but has side effects such as nausea, vomiting and headache.^194^ There are mainly four subtypes of PDE4, A to D. In recent years, the anti-inflammatory effect of PDE4 inhibitors has been shown to be mainly related to PDE4B while PDE4D is related to side effects such as digestive tract symptoms.^187^ The development of new PDE4B subtype-specific agents is expected, and inhalants that reduce systemic side effects are also being developed. In addition, oher PDE4 inhibitors is being developed now^195–198^ (Table 3).

### Endothelin inhibitors

Endothelin signalling plays a major role in pulmonary hypertension secondary to COPD. Endothelin antagonises bosentan (treatment for 18 months) can alleviate the condition of COPD patients and prevent the progression of pulmonary hypertension. This effect is more significant in GOLD III and IV patients.^199^ But for COPD patients without pulmonary hypertension, bosentan will aggravate their hypoxaemia.^200^ At present, further research on the role of bosentan in patients with GOLD III or IV COPD and pulmonary hypertension in the acute exacerbation phase is ongoing, but its status is unclear^201^ (Table 3).

### Vasoactive intestinal peptide

Vasoactive intestinal peptide (VIP) has been characterised as a vasodilatory peptide.^202^ It exerts a wide range of biological actions, such as bronchodilation, anti-inflammatory effects,^203^ via binding its receptor VPAC1 or VPAC2 to increase significantly cAMP,^204^ adenylate cylase^205^ and phospholipase C,^206^ which cause different downregulation on a variety of transcription factors. VPAC1 receptor mRNA is abundant in lung and T lymphocytes. VPAC2 receptor is mainly distributed in the smooth muscle layer and the base of mucosal epithelium in lung. VPAC1 was particularly elevated in alveolar macrophages of COPD patients.^207^ VPAC2 receptor was activated by VIP, and inhibited the CSE-induced cytotoxicity of rat lung alveolar L2 cells.^208^ Increased serum VIP levels are associated with acute exacerbation of COPD patients.^209^ VIP has significant therapeutic potential in the treatment of COPD.^208^ How, it clinical application might be limited because of the short half-life of plasma after intravenous administration and the difficulty of routes.^210,211^ VIP (3-month inhaled treatment) was performed in severe COPD patients. Study was completed in 2006 but no results are available^201^ (Table 3).

### Adenosine A_2a_ receptor agonist

Adenosine is a natural purine nucleoside that is ubiquitous in human tissues and plays a key role in many biological processes, such as energy production and protein metabolism.^212^ At present, four subtypes (A_1_, A_2a_, A_2b_ and A_3_) of adenosine receptors have been cloned. The anti-inflammatory effect of adenosine is mainly attributed to occupancy of cAMP-elevating Gs-protein-coupled A2a-receptors.^213^ The key molecular mechanism is the suppression of NF-κB pathway activated by cytokines such as TNF-α and IL-1β. A_2a_ receptor plays an anti-inflammatory role in specific cells and various inflammation models.^214^ Knockout of the A_2a_ receptor gene will cause mucus production, airway destruction and lung inflammation.^215^ Currently, several adenosine A_2a_ receptor agonists have been proven effective in COPD models, but there are cardiovascular side effects.^214^ A Phase 4, randomised, double-blind study (NCT00862641) assessed the safety of the selective adenosine A_2a_ receptor agonist, regadenoson, compared with placebo in subjects with asthma or COPD, and the result showed randomised did not modify repeated forced expiratory volume in 1 s (FEV1) when compared to placebo.^216^ UK432,097, which is beneficial in the lungs of anaesthetised guinea pig without any obvious cardiovascular side-effects.^217^ Phosphorylated A_2a_ receptor agonists are under development to reduce adverse effects such as hypotension^218^ (Table 3).

### Macrolides

Macrolide antibiotics are secondary metabolites of a variety of Actinomycetes bacteria. The molecule contains a 14–16-membered macrolide structure, which has a wide range of functions. It has not only antibacterial function, but also anti-inflammatory effect, inhibition of mucus secretion and immune regulation.^219,220^ The anti-inflammatory and immunomodulatory functions are mainly 14-ring and 15-ring.^221^ Clinically, they are used for long-term treatment of chronic inflammatory lung diseases such as COPD.^222^ The anti-inflammatory mechanism of macrolide antibiotics is complex and has not been fully elucidated. It may reduce the inflammation of COPD by regulating the PI3K/Akt-Nrf2 pathway and control transcription factors such as NF-κB and AP-1 to inhibit the production of inflammatory cytokines.^140^ Animal models provided further evidence that clarithromycin has an inhibitory effect on the development of emphysema, and its dose is almost the same as the clinical dose.^223^ The potential benefit of a new antibiotic, solithromycin was studied for the long-term treatment of COPD, but due to the early termination of the study, there were too few subjects and data collected to perform statistical analysis. Other macrolides without anti-bacterial activity are being developed as anti-inflammatory drugs and clinical trials are expected in the future (Table 3).

### PPAR agonists

Peroxisome proliferator activated receptors (PPARs) are ligand-activated nuclear hormone receptors belonging to the steroid receptor superfamily, including three recognised subtypes (PPARα, PPARγ and PPARδ). The activation of PPARγ and PPARα may have anti-inflammatory and immunomodulatory effects.^224^ PPARγ exert antioxidant and anti-inflammatory effects by down-regulating NF-κB and other pro-inflammatory transcription factors.^225^ Cigarette smoke will down-regulate the expression of PPARγ, and the level of PPARγ in the lung tissues of COPD patients is significantly lower than that of normal people.^226^ PPARγ agonists, such as the thiazolidinediones, rosiglitazone and pioglitazone, have been shown to reduce lung inflammation in mouse models of tobacco smoke, and studies have found that treating model mice with thiazolidinedione can reverse emphysema.^227,228^ In addition, PPARγ agonists can also inhibit pulmonary fibrosis, which is expected to be a drug to prevent small airway fibrosis in COPD.^229^ A retrospective epidemiological study showed that diabetes patients treated with PPARγ agonists have a significantly reduced risk of COPD exacerbation, but their risk of cardiovascular risk events has also increased.^230^ In addition, Only large doses of thiazolidinediones can produce anti-inflammatory effects, which leads to speculation about the correlation of PPARγ stimulation in COPD.^231^ Non-thiazolidinedione PPARγ ligands are currently being studied to reduce potential cardiovascular risks. PPARα agonists, such as fenofibrate, may have therapeutic potential in the treatment of systemic symptoms of COPD (Table 3).

### NF-κB inhibitors

A Japanese Drug Discovery Company is developing a compound code named IMD-1041, which is an IKK β inhibitor developed for the treatment of COPD, but has no follow-up information posted since April 2009. It is unclear whether study was performed. BMS-345541 is a highly selective IKK inhibitor with good pharmacokinetic characteristics. In the human airway smooth muscle cells, co-incubation with BMS-345541 markedly inhibited the NF-κB nuclear translocation induced by TNF-α and IL-13.^232^ PS-1145 induce a dose-dependent inhibition of phosphorylated IkBα and NF-κB activation, and then reduces the expression of adhesion molecules, cytokines and chemokines on airway smooth muscle cells.^233^ A small molecule IKK2 inhibitor is under development as a therapy for inhibiting the NF-κB activity.^234^ The effectiveness of IKK2 inhibitors has been verified in animal models of COPD;^235^ and clinical trials of IKK2 inhibitors in patients with bronchial asthma and joint rheumatism have also been conducted. IKK2 inhibitors are expected to be used as a new therapeutic drug for COPD in the future following in-depth research. Further developments include NF-κB “decoy” oligonucleotides and antisense and small interfering RNA agents.^236,237^ In addition, NF-κB-deficient mice have been reported to be more prone to sepsis,^238^ hence complications such as immunosuppression and infection susceptibility caused by long-term NF-κB inhibition must be considered (Table 4).

**Table 4:** Development of proinflammatory signalling pathway inhibitors for COPD

Drug	Mechanism/effect	Clinical progress	Reference
IKK inhibitor	Inhibition of IKK in NF-κB pathway	IMD-1041 has no follow-up information posted since April 2009. It is unclear whether study was performed (NCT00883584). BMS-345541 and PS-1145 has no approvals for human/medical use or for use in clinical trials. No clinical trials mention it. The information id from the U.S. National Library of Medicine.	^341^
P38 MAPK inhibitor	Inhibition of p38MAPK pathway	SB-681323 significantly reduced TNF-α production in COPD (NCT00144859) but the study was discontinued. PH-797804 (6-week treatment) (NCT00559910) significantly improved lung function and dyspnoea in moderate-to-severe COPD in DBPCRT but was discontinued. RV568 (14-day inhaled treatment) significantly increased FEV1 and reduced sputum malondialdehyde and serum myeloperoxidase in COPD patients. However, a recent conference report showed that 12 weeks of rv568 treatment had no benefit for lung function in more than 200 COPD patients, (NCT01867762, NCT01475292, and NCT01661244).	^246,342,343^
Nemiralisib (GSK2269557)	Inhibition of PI3K	Clinical studies on TG100-115 and AS605240 are required. GSK2269557 (NCT02522299 for 84 days or NCT02294734 for 28 days) was used in patients with acute exacerbation of COPD in progress of DBPCRTs RV1729 (up to 28 days of treatment) is being tested at NCT02140346 and limited efficacy data have been collected in major phase I studies.	^344^
VIP	Significantly increase cAMP, adenylate cylase and phospholipase C	VIP (3-month inhaled treatment) was performed in severe COPD patients. Study was completed in 2006 but no results are available (NCT00464932).	^345^
Adenosine A2A receptor	exert anti- inflammatory effect by enhancing cAMP	Regadenoson group (2 months) occurred with higher incidence of Dyspnoea, and unable to modify repeated FEV1 when compared to placebo ((NCT00862641). UK432,097 is beneficial in the lungs of anaesthetised guinea pig without any obvious cardiovascular side-effects. But UK-432097 (6-week inhaled treatment) in DBPCRT showed no significant improvement in FEV1 and quality of life parameters (NCT00430300).	^216,346,347^

### p38MAPK inhibitors

As a new type of anti-inflammatory drug, p38MAPK inhibitors have attracted much attention from researchers. Currently, various small-molecule p38MAPK inhibitors have been developed and verified in animal models of smoking-induced pneumonia, proving their beneficial anti-inflammatory effects.^239^ Inhibiting p38MAPK activation has been found to reduce the CS-induced airway smooth muscle cell proliferation,^240^ suggesting that p38MAPK inhibitors may reduce the progression of COPD airway remodelling. p38MAPK inhibitors also reduce cytokine production by alveolar macrophages.^241^ In addition, there is evidence that corticosteroids cannot inhibit p38MAPK activation, and that p38MAPK inhibitors combined with corticosteroids enhance the inhibitory effect of corticosteroids on cytokines produced by macrophages in patients with COPD mediated by LPS.^239^ p38MAPK inhibitors have a unique advantage in patients with a poor hormone response. A 28 days trial of p38MAPK inhibitor SB681323 in patients with moderate stable COPD reduced sputum neutrophils and plasma fibrinogen with improvement in forced vital capacity.^242^ The patients with moderate to severe COPD receiving p38MAPK inhibitor PH797804 for 6 week decreased serum CRP levels, and induced a significant increase of FEV1 and a concomitant improvement in dyspnoea score.^243^ Each subtype of p38MAPK has unique functions due to differential expression across tissue types and different regulatory kinases and downstream genes, hence their targeting comes with adverse effects. Although some clinical trials are in progress, due to severe side effects such as those caused by an undesired pharmacological activity, suppression of the innate immune response to viral and bacterial infections, and damage to the central nervous system and liver,^244,245^ these drugs remain challenging for a clinical application. There is a need to develop inhaled formulations and selective inhibitors of the α-δ subgroups. The inhaled narrow spectrum kinase (p38α + Src family) inhibitor (JNJ49095397/RV568) in patients with moderate to severe COPD decreased serum CRP levels as well as improved trough FEV1 and dyspnoea index scores.^246^ However, p38αMAPK inhibitors block the upstream MAPK kinase kinases, leading to hyperactivation of the transforming growth factor-activated kinase-1 and mixed-lineage kinase which then hyperactivate the JNK. Therefore, other drugs that target more downstream substrates should be also developed (Table 4).

### PI3K inhibitors

PI3K is divided into three categories, namely classes I, II and III, among which class I PI3K is most widely studied. Class I PI3K is a heterodimer composed of a regulatory subunit (p85) and a catalytic subunit (p110).^247^ There are four types of catalytic subunits: p110α, p110β, p110δ and p110γ, and while δ and γ subunits are limited to white blood cells, α and β subunits are widely distributed in various cells.^248^ PI3K, especially PI3K δ and γ subtypes, are closely related to a COPD inflammation. PI3K inhibitors reduce nitric oxide production by inhibiting carbon monoxide synthase.^249^ Studies have shown that interruption of the PI3K pathway improves severe COPD protease imbalance.^250^ Aerosolized TG100-115, a compound that selectively blocks PI3Kγ and PI3Kδ, inhibits pulmonary neutrophils induced by intranasal LPS and smoke in mice with chronic obstructive pulmonary disease.^251^ AS605240 is a selective inhibitor of PI3Kγ, which reduces the migration of polymorphonuclear leucocytes in vitro and the infiltration of polymorphonuclear leucocytes in the lungs of mice with LPS induced lung injury.^137^ The interventional therapy of TG100-115 was successful even in steroid resistant COPD induced by smoking in mice.^251^ Various PI3K inhibitors are currently being used in clinical trials, primarily for malignant tumours.^252,253^ In recent years, inhaled PI3Kγ/δ inhibitors have been reported to inhibit pneumonia caused by smoking in animal models and have been especially effective and safe for patients with glucocorticoid contraindications.^254^ Specific PI3Kδ inhibitors, GSK2269557 and RV1729 are being developed,67 and studies on the effects of such inhibitors in COPD are in progress. Efficacy data remain limited.^255,256^ In contrast, even selective P13K subtype inhibitors have the risk of immunosuppression and secondary bacterial infections,^251^ and reducing the occurrence of side effects will be an important issue (Table 4).

## Trx and its effect in COPD

Trx is a multifunctional protein consisting of 105 amino acids with a molecular weight of 12 kDa and a highly conserved Cys-Gly-Pro-Cys active site. Trx exists in two forms: oxidised (Trx-S2) and reduced (Trx-(SH)2). Trx-S2 can be reduced to Trx-(SH)2 by the exchange reaction of -SS- and -SH under the action of thioredoxin reductase (TrxR) and nicotinamide adenine dinucleotide phosphate (NADPH).^257^ Trx cooperates with TrxR and NADPH to form the Trx system. Trx catalyses the reduction of disulphide bonds and quenches ROS by coupling with Trx-dependent peroxidases or peroxiredoxins. In addition to its antioxidant effects, Trx has a crucial role in the redox regulation of cellular signalling and activation. Trx is involved in various redox-dependent cellular processes such as gene expression, signal transduction, cell growth, apoptosis and interacts with various target molecules.^258,259^ Under stress conditions, Trx is released into the extracellular space where it exerts a cytoprotective effect and cytokine-like activities.^260^

Trx expression in the sputum of COPD patients is positively correlated with the degree of hypoxia.^261^ Mice that overexpress human Trx can effectively inhibit a CS-induced emphysema and pulmonary inflammation.^262^ Intraperitoneal injection of Trx suppress a smoke-induced murine pulmonary inflammation by inhibiting the production and release of cytokines, inflammatory mediators, chemokines and ROS.^263^ Trx inducer increases the Trx expression in murine lung tissue and improves lung injury.^261^ Recent research has also shown that inhaled Trx also reduces a smoke-induced chronic lung injury. Currently, clinical trials targeting acute lung diseases have entered the preparation stage. At the same time, as a pre-clinical trial of COPD, intravenous infusion therapy for acute exacerbations, protein inhalation therapy for stable phase, and oral administration of inducers are also underway.

## Trx in COPD treatment

### Adjusting the redox balance

Trx plays an important role in maintaining the body’s redox balance. Trx can be used as an electron donor to reduce H_2_O_2_ and tertiary butyl hydroperoxide. In addition, when the body’s peroxidase reduces hydrogen peroxide, Trx is also needed to provide a certain reduction equivalent.^264^ Further, there are other redox systems similar to the Trx system in the body, such as the glutathione (GSH) system.^261^ The Trx system and the GSH system jointly control the redox system. The equilibrium state of the environment, and the Trx and GSH systems cross each other to provide electrons and serve as a compensation system.^265,266^ In addition, Trx and thioredoxin-interacting protein (TXNIP), a negative regulator, constitute a redox-like protein compound (redoxisome) that regulates a variety of redox-sensitive signals and is essential for maintaining the intracellular and extracellular redox balance and monitoring inflammatory responses.^267^ Without an oxidative stress, TXNIP is in a bound state with Trx. When the intracellular ROS content increases, Trx and TXNIP are dissociated, and Trx bind to ROS. Dissociated TXNIP combines with and activates the NLRP3 inflammasome to induce IL-1β expression in a NLRP3-ASC-caspase-1-dependent manner, thus causing inflammatory reactions.^266^ This signalling complex may be a key regulatory mechanism for the body to regulate cellular redox balance and prevent the progression or exacerbation of stress-induced diseases.^268^

### Regulating the protease balance

Both endogenous and exogenous Trx inhibit and improve a protease-induced emphysema.^269^ MMP-9 and MMP-2 play important roles in COPD. Oxidative stress upregulates the MMP-9 expression while Trx inhibits MMP-9 via its antioxidant properties.^270,271^ The mechanism may be inhibition of p38MAPK and JNK.^272^ In addition to inhibiting MMP, Trx also inhibits its inhibitor, TIMP. Studies have found that Trx has a differential inhibitory effect on MMP-9, MMP-2, TIMP-1 and TIMP-2, thereby regulating the MMP/TIMP balance.^273^ Trx suppresses MMP and TIMP by reducing their activity but not degrading TIMP and MMP.^274,275^ The activity of TIMP-1, TIMP-2 or MMP-2 was not inhibited by a version of Trx lacking a disulphide reductase activity or Trx with a TrxR deficiency.^274,276^ This indicates that Trx regulates the MMP/TIMP balance by differentially inhibiting the activities of TIMP and MMP, and this process depends on the redox active sites of Trx and TrxR.

### Regulating inflammatory cells, cytokines and chemokines

Trx inhibits the migration and activation of inflammatory cells such as neutrophils, reduces the release of inflammatory factors, and reduces the inflammatory response. Both in vivo and in vitro studies have shown that Trx inhibits the chemotaxis of macrophages, lymphocytes, and neutrophils.^253,277^ During COPD development, the neutrophils and alveolar macrophages are activated to produce various inflammatory mediators, including IL-1β, IL-6, IL-8 and TNF-α. Trx significantly inhibits the production and release of IL-1β, IL-6, IL-8 and TNF-α induced by LPS in the human monocyte-derived macrophages. This is achieved by Trx blocking the NF-κB pathway or inhibiting an inflammatory signal activation by cell surface molecules.^240,278^ The specific mechanism of action has been thoroughly explained in our previous article.^279^

### Regulating adhesion factors and growth factors

L-selectin (CD62L) is a shedding molecule on neutrophils that plays a vital role in guiding neutrophils to adhere to the vascular endothelium and penetrate the blood vessels. Trx inhibited the LPS-induced downregulation of L-selectin exfoliation on neutrophils and reduced the L-selectin attachment to endothelial cells whereas double-mutant Trx C32S/C35S did not inhibit neutrophil adhesion to the endothelial cells.^280^ In a rat model of LPS-induced inflammation, systemic Trx significantly reduced neutrophil infiltration in the bronchus and lung tissues, but did not directly reduce the increased LPS-induced ICAM-1 present on the endothelial cells.^281^

Trx undergoes oxidation in response to EGF.^282^ The local and specific oxidation of Trx occurs during the ROS signalling produced by EGF stimulation.^283^ EGFR signal transduction requires a special endoplasmic reticulum Trx to control receptor expression on the cell surface.^257^ Intracellular Trx inhibits EGFR synthesis and activation. TGF-β1 activates Smad2/3, PI3K/Akt, ERK1/2, GSK-3β and/or p38MAPK signalling to induce pulmonary fibrosis and EMT.^284,285^ Trx inhibits the MPK38-induced TGF-β function in a phosphorylation-dependent mannerm.^286^ Trx inhibits bleomycin-induced skin fibrosis by down-regulating TGF-β.^287^ Trx overexpression inhibits airway remodelling by inhibiting TGF-β1 and EGFR.^288^

### Regulating cAMP

cAMP plays a protective role in COPD inflammation through its effector proteins EPAC and PKA. In animal models of brain injury, Trx protected the astrocytes by activating cAMP-PKA and inhibiting astrocyte apoptosis.^289^ Down-regulating the Trx gene inhibits the cAMP-PKA pathway to cause apoptosis, exacerbating astrocyte damage caused by an oxygen-glucose deprivation/reoxygenation in vitro.^290^ Trx is necessary for the nerve growth factor (NGF) signalling through its cAMP responsive element to drive expression of c-fos, which has been hypothesised to be critical for the function of NGF.^289^ PKA activity is inhibited by the hydrogen peroxide formed by insulin but activated by Trx, which restores the newly formed -SS- bond of PKA to the -SH group.^291^ We suggest that Trx may also protect lung cells by acting on the cAMP-PKA pathway in COPD.

### Regulating the NF-κB and MAPK pathways

Trx suppresses the inflammatory response by regulating the NF-κB and MAPK pathways. Trx modulates NF-κB activity and plays different roles extracellularly and intracellularly. Inhibiting the nuclear Trx blocks the nuclear activities of NF-κB and AP-1 dependent transcription factors and reduces neutrophil invasion and TNF-α production.^292^ Extracellular Trx inhibits the production of p50 and p65 and promotes IκB synthesis by acting on cell membrane surface receptors to limit NF-κB activation and translocation into the nucleus, thereby blocking the NF-κB pathway.^293^

Trx inhibits eotaxin-induced phosphorylation of extracellular signal-regulated kinase 1/2 and p38 by reducing the activation of ERK1/2 and p38MAPKs.^294^ Under normal conditions, Trx binds to the N-terminal region of the apoptosis signal-regulating kinase 1 (ASK1). ASK1 is a member of the MAPK kinase family and elicits a wide variety of cellular responses to various types of stress by activating the JNK and p38MAPK pathways. Under oxidative stress, Trx separates from ASK1, activating ASK1.^295^ This indicates that Trx acts as an upstream inhibitor of ASK1, and Trx/ASK1 signalling is an upstream modulator of p38MAPK.^296^ Trx suppresses p38MAPK activation in the LPS-stimulated neutrophils.^280^ In addition, ROS exacerbates airway inflammation by activating inflammatory mediators and transcription factors, such as NF-κB, MAPK and AP-1.^297^ This suggests that Trx regulates the p38MAPK pathway by removing ROS.

### Adjusting the PI3K/Akt signalling pathway

The PI3K/Akt signalling pathway is a classic signalling channel that can be activated by various extracellular signals including growth factors, cytokines, and oxidative stress, and plays an important role in COPD. Trx inhibits the indomethacin-induced ROS production and inhibits the expression of phosphorylated Akt in rat gastric epithelial cells.^298^ In addition, Trx deficiency reduces the expression of Akt1 and pAkt by NO.^299^ The main activation signals of PI3K/Akt signalling are inhibited by Trx, and downstream Akt phosphorylation was also inhibited by Trx. Therefore, we suggest that Trx likely plays an important role in the PI3K/Akt signalling pathway.

### Improving glucocorticoid resistance

**Fig. 3:**
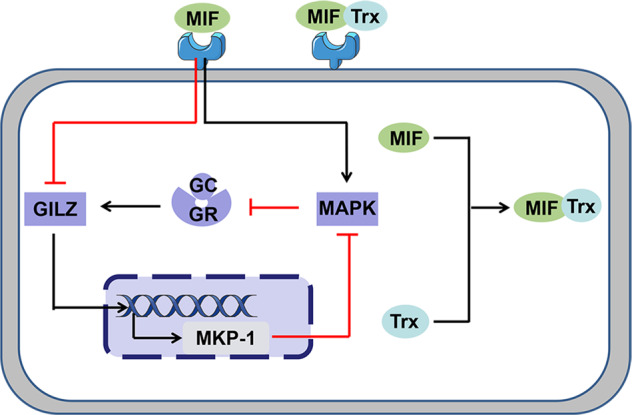
Trx improves GC through MIF. One GC resistance mechanism impaired by the MIF is the loss of GC sensitivity via inhibition of MKP-1. MKP-1 is induced by GC to mediate GC inhibition of ERK, JNK and p38MAPK activities and cytokine production. MIF inhibits GILZ expression via a unique set of effects on transcription factor expression and phosphorylation. MKP-1 and MAPK activation are regulated by MIF via GILZ. Both intracellular and extracellular Trx bind to MIF and form a heterodimer to prevent the MIF entry into cells and MIF-induced glucocorticoid resistance

COPD is relatively resistant to modulation by corticosteroids; even high doses of glucocorticoid (GC) do not delay or inhibit COPD progression.^300^ One mechanism of GC resistance is the impairment of GC sensitivity by the macrophage migration inhibitory factor (MIF) via MAP kinase phosphatase-1 (MKP-1) inhibition.^300^ MIF is part of a class of pleiotropic immunomodulatory factors with a unique structure that functions similar to chemokines and promotes inflammatory responses, directed cell migration, and release of other cellular inflammatory factors.^301^ MIF may play a key role in the pathogenesis of airway inflammation.^302–304^ MIF has at least two catalytic activities: tautomerase and oxidoreductase activities. The oxidoreductase activity of MIF is closely related to the Trx family.^301,305–307^ MKP-1 is an archetypal member of the dual specificity phosphatase family that inactivates the MAPK activity in response to pro-inflammatory stimuli.^308–310^ MKP-1 is induced by GC to mediate GC inhibition of ERK, JNK and p38MAPK activities and cytokine production induced by pro-inflammatory stimuli such as LPS or IL-1.^311–313^ It has recently been demonstrated that MIF inhibits the GC-induced leucine zipper (GILZ) expression via a unique set of effects on transcription factor expression and phosphorylation, and regulation by MIF of MKP-1 and MAPK activation are mediated through GILZ (Fig. 3).^314^

Trx regulates MIF expression levels and inhibits inflammation caused by MIF.^315,316^ In a mouse asthma model, transgenic overexpression of Trx significantly reduced MIF expression in the airway epithelial cells and reduced the number of MIF-positive inflammatory cells in the lung parenchyma.^260^ Trx inhibits MIF production in human monocytes.^317^ MIF has a specific affinity for Trx; extracellular MIF is internalised into cells exclusively by binding to Trx on the cell membrane. Exogenous Trx and intracellular Trx combine with MIF to form a complex which affects the MIF-induced inflammatory response.^318^ In addition, some studies have also demonstrated that Trx directly bind to glucocorticoid receptor and enhance the cell’s response to glucocorticoids.^319,320^ Although there are not the evidence showing the effect of Trx on HDAC2, we suppose that Trx may increase HDAC2 activity by regulating cellular redox signalling, and we would like to prove this attractive hypothesis in our next studies.

### Effects on the immune system

Trx has no inhibitory effect on the immune system while regulating inflammatory responses in various inflammation models.^317,321,322^ There is no significant difference in the population and differentiation of immune cells such as the mast cells, dendritic cells, and lymphocytes between Trx overexpression and WT animals.^323^ Oxidative stress promotes the Th2 type immune response by inducing Th1 cell apoptosis and Th2 cell differentiation, breaking the Th1/Th2 balance, and causing Th2 airway inflammation.^324,325^ After the OVA challenge, IL-13 expression in BALF of Trx-Tg mice was significantly lower than that in WT mice, while serum levels of IL-13 were comparable.^260^ This shows that Trx inhibits local Th2 cells to push the balance towards Th1 and suppress local inflammation while having no effect on the Th1/Th2 balance of the systemic immune system. Bronchial LN (BLN) cells isolated from the Trx-Tg mice produced an equal amount of Th2 cytokines IL-4, IL-5 and IL-13 as the BLN cells of WT mice after leaving the high Trx environment.^240^ This shows that Trx does not directly affect the Th1/Th2 proliferation and differentiation, but rather inhibits inflammation by regulating the production and release of Th1/Th2 cytokines.

## Conclusions and perspectives

**Fig. 4:**
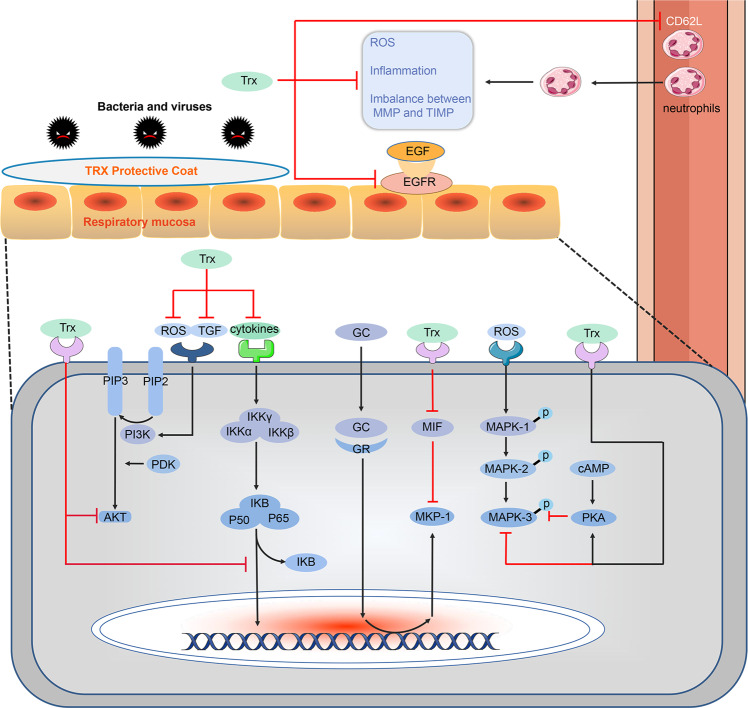
Trx prevents and relieves COPD pathogenesis through multiple molecular mechanisms. Trx eliminates MIF to improve glucocorticoid resistance and eliminates ROS and inhibits neutrophil infiltration by regulating adhesion molecules to suppress the production of cytokines to reduce oxidative stress and inflammation. Trx exerts its anti-oxidative and anti-inflammatory effects by regulating the NF-κB, MAPK, PI3K/Akt and cAMP-PKA pathways. Trx also inhibits the airway neutrophil recruitment by down-regulating the expression of neutrophil L-selectin on circulating neutrophils. Trx is subtle in regulating the balance between protease and antiprotease. Trx has inhibitory effect on both, but it is asymmetric in its inhibition. Trx has stronger inhibitory effects on over-generated proteases, thus maintaining the balance of the protease–antiprotease system. Moreover, Trx down-regulates the expression of EGFR and TGF in the airway to reduce mucus secretion, airway remodelling and pulmonary fibrosis

COPD pathogenesis is mainly related to the overexpression of inflammatory mediators and cytokines, the activation of inflammatory signalling pathways, the protease/anti-protease imbalance, and the oxidation–antioxidation imbalance. These factors are interrelated and it is difficult to achieve the desired treatment results through a single target. Owing to the overlapping function of molecular targeted inflammatory signals, the degree to which pathogenesis of COPD can be prevented if only one pathway is inhibited remains unknown. At present, some drugs have been proven to be effective in animal tests; some are challenging to be used in clinical trials due to significant side effects while others continue to be in the hypothetical stage and have not been proven effective in treating COPD. Therefore, further studies of the functions and mechanisms of various target molecules are necessary, and their effectiveness and safety through must be evaluated through animal experiments and clinical trials. Trx plays an important role in the treatment of COPD (Fig. 4). Its mechanism of action is highly unified with the pathogenesis of COPD, and it effectively inhibits the occurrence and development of COPD through a variety of mechanisms. Trx also improves the GC resistance of COPD. Whether used as a supplement to existing therapies or for patients with poor response to hormones, Trx has unique advantages. Therefore, we suggest that Trx has good prospects in treating COPD and may be an important drug for COPD treatment in the future.
